# Serious juvenile offenders: classification into subgroups based on static and dynamic charateristics

**DOI:** 10.1186/s13034-017-0201-4

**Published:** 2017-12-22

**Authors:** Sanne L. Hillege, Eddy F. J. M. Brand, Eva A. Mulder, Robert R. J. M. Vermeiren, Lieke van Domburgh

**Affiliations:** 1 Department of Child and Adolescent Psychiatry, VU University Medical Center, Duivendrecht, P.O. Box 303, Amsterdam, 1115 ZG The Netherlands; 2 Intermetzo-Pluryn, Nijmegen, The Netherlands; 3 Department of Justice, National Agency of Correctional Institutions, The Hague, The Netherlands; 4 Curium-LUMC, Leiden University Medical Center, Leiden, The Netherlands

**Keywords:** Serious juvenile offenders, Risk factors, Cluster-analysis, Subgroups

## Abstract

**Background:**

The population in juvenile justice institutions is heterogeneous, as juveniles display a large variety of individual, psychological and social problems. This variety of risk factors and personal characteristics complicates treatment planning. Insight into subgroups and specific profiles of problems in serious juvenile offenders is helpful in identifying important treatment indicators for each subgroup of serious juvenile offenders.

**Methods:**

To identify subgroups with combined offender characteristics, cluster-analyses were performed on data of 2010 adolescents from all juvenile justice institutions in the Netherlands. The study included a wide spectrum of static and dynamic offender characteristics and was a replication of a previous study, in order to replicate and validate the identified subgroups. To identify the subgroups that are most useful in clinical practice, different numbers of subgroup-solutions were presented to clinicians.

**Results:**

Combining both good statistical fit and clinical relevance resulted in seven subgroups. Most subgroups resemble the subgroups found in the previous study and one extra subgroups was identified. Subgroups were named after their own identifying characteristics: (1) sexual problems, (2) antisocial identity and mental health problems, (3) lack of empathy and conscience, (4) flat profile, (5) family problems, (6) substance use problems, and (7) sexual, cognitive and social problems.

**Conclusions:**

Subgroups of offenders as identified seem rather stable. Therefore risk factor scores can help to identify characteristics of serious juvenile offenders, which can be used in clinical practice to adjust treatment to the specific risk and needs of each subgroup.

**Electronic supplementary material:**

The online version of this article (10.1186/s13034-017-0201-4) contains supplementary material, which is available to authorized users.

## Background

The population of serious juvenile offenders in Juvenile Justice Institutions (JJIs) is heterogeneous in its background, mental health issues, offending behavior and attitude towards treatment [1, 2]. Serious juvenile offenders often display problems in several life areas that all impact daily functioning and show risk factors on different domains. Therefore, the potential number of different combinations of risk factors in individuals is substantial. So far, many studies on characteristics of serious juvenile offenders are based on the population as a whole and do not take the heterogeneity within this population into consideration. However, given their heterogeneity, findings based on overall group statistics cannot automatically be used in individual clinical treatment planning and therefore leaves a gap between science and practice [3]. Identifying subgroups of serious juvenile offenders in the larger population may help to find more specific treatment indicators for more homogeneous subgroups of individuals. This is a step towards the development of individualized treatment for these juveniles.

The main objectives of treatment of serious juvenile offenders in JJIs are to reduce criminal recidivism, to prevent further harm to society, and to create a positive future on different domains for the individual. Well-known theoretical frameworks such as the Risk Needs and Responsivity model (RNR) [4] and the Good Lives Model (GLM) [5] state that treatment works best when tailored to specific individual characteristics. Based on the RNR model, the intensity of treatment has to be adjusted to the level of *risk* and interventions should aim at the *needs* related to criminogenic factors. According to the *responsivity* principle, interventions should also match the offenders personal characteristics, such as learning style and motivation. Several studies demonstrate that the number of risk factors are more predictive of reoffending behavior, than one particular risk factor [6]. Hence, information about characteristics related to these three elements is needed in order to work on reducing recidivism. However, within forensic psychiatry, clinicians not only focus on recidivism reduction, but also on treating individuals with mental health problems. Therefore clinicians constantly have to find a balance between protecting the society against ‘offenders’ and providing care for ‘patients’ [7]. Forensic practitioners have therefore previously been described as ‘double agents’ using different objectives when developing treatment plans [8]. Since recent studies demonstrate high prevalence rates of chronic and comorbid mental health problems [2, 9–11], cognitive impairment [12], and trauma [13] in incarcerated adolescents, these offender characteristics should be integrated in treatment as well. This in order to provide good care and to create optimal circumstances for treatment and development for the individual serious juvenile offender. Thus, problems that are not directly linked to criminal behavior or recidivism, need to be taken into account during individualized treatment planning as well.

In everyday practice, it is challenging to integrate these different models, and to design individual treatment trajectories considering all possible risk factors and offender characteristics for each of the serious juvenile offenders in care. To support clinicians in this process, it will help to identify subgroups with a common pattern of risk factors within the group of serious juvenile offenders. If clinicians are able to choose interventions matching the specific needs of a subgroup a juvenile belongs to, a next step will be taken towards individualized treatment. Thus, knowledge is needed on which subgroups can be recognized based on clustering of risk factors and which risk factors point towards treatment indicators within these subgroups. Classification of a larger population into subgroups also enables clinicians to learn from previous experiences and to study treatment interventions for specific subgroups of serious juvenile offenders.

For decades, the population of serious juvenile offenders has been studied and classifications of this heterogeneous group have been developed [12, 14, 15]. So far, most studies on subgroups of serious juvenile offenders have used offending behavior [16, 17] or the severity, nature, and chronicity of the careers of the offenders [6] to distinguish subgroups. Characteristics of the serious juvenile offenders that are considered important for treatment according to the above mentioned models, such as motivation for treatment, cognitive skills and attitude in the institution together with mental health issues, are not included in these studies on typologies of serious juvenile offenders. Studies that did focus on mental health issues in serious juvenile offenders [1, 18–20], or on gender [21, 22] mainly focused on specific subgroups of offenders without making comparisons *between* subgroups of serious juvenile offenders. In addition, these studies focused on relatively small populations, which makes it impossible to identify clear subgroups and provide clinicians with valuable information. As a result, data on the uniqueness of offender characteristics, other than offense characteristics, for specific subgroups of offenders, is lacking. To overcome these limitations, Mulder, Brand, Bullens, and van Marle identified subgroups of offenders based on a wide variety of risk factors in a nation-wide sample of incarcerated youth [23]. This study of Mulder and colleagues identified subgroups based on data driven research which provided certain fit values, combined with the face value after the consultation of experts in the forensic field. Six subgroups with different risk profiles were found, named: (1) antisocial identity, (2) frequent offenders, (3) flat profile, (4) sexual problems and weak social identity, (5) sexual problems, and (6) problematic family background [23]. Since the identification of subgroups by algorithms is an exploratory heuristics process that can create as well as reveal structure, replication is critical to establish validity [24]. Besides replication, the clinical value of the subgroups would improve when more insight is provided about differences and resemblances in risk factors between the identified subgroups on an item level, as this could inform clinical intervention strategies. Therefore, the present study aims to replicate the previous study by Mulder and colleagues and to study the subgroup characteristics on item level.

Using cluster-analyses, the present study identifies subgroups within a nationwide population of serious juvenile offenders from JJIs. We are interested in the identification of subgroups in the total JJI population, including male and females. A sample twice as large as the original sample was used with information on offender characteristics, including a wide variety of static and dynamic risk factors and mental health problems. In order to identify the solution with the highest clinical relevance, different subgroup solutions and their risk profiles were discussed with clinicians. Finally, the present study takes the identification of the subgroups one step further by taking a more detailed look at the differences between subgroups on item level of the different risk factors. These analyses result in combinations of distinguishing offender characteristics per subgroup, that enables clinicians to tailor treatment to individual needs according to the principles of prevailing theories on offender treatment and create optimal treatment circumstances per individual.

## Methods

### Subjects

The subjects of this study were adolescents aged 12–22 years and sentenced with a mandatory treatment order in a JJI in the Netherlands between January 1994 and December 2013. This mandatory treatment order (PIJ, Placement in Juvenile Justice Institution) [25] is the most severe measure in the Netherlands and is intended for adolescents between the age of 12 and 22 who committed a severe crime and have a mental disorder or deficient (emotional or cognitive) development [26]. The mandatory treatment order initially lasts 2 years, but can be extended to 4 or 6 years in case of insufficient development concerning risk factors and reintegration.

The total sample included 2010 adolescents and represented the most serious offenders in the Netherlands. The majority (95%, n = 1911) was male and only 5% (n = 99) was female and both genders were included since the interest of the present study was on the total population of serious juvenile offenders in the JJIs. The background and characteristics (age by start treatment order, IQ, and origin of offenses) of both genders did not differ significantly, therefore both genders were included in the current study. The mean age at the start of the treatment order was 17.0 years (SD 1.46), 4.6% was 14 years or younger, and only 1.4% was older than 20 at the start of the treatment order. The offenses leading to the mandatory treatment order were violent offense (58.7%), sexual offenses (25.6%), and (repeating) property offenses (15.7%). In line with policy of the Dutch Ministry of Safety and Justice, no information about ethnicity was collected. The study of Mulder and colleagues [23] included 1107 adolescents, which are also included in the current sample.

### Instruments

#### Juvenile Forensic Profile (JFP)

We used a list of 70 items specially constructed for forensic research based on file information, the Juvenile Forensic Profile (JFP) [27]. This list of items was developed in 2003 and 2004 and contains items similar to items in internationally and nationally validated instruments for risk assessment together with instruments for measuring problem behavior, including the Child Behaviour Check List [28], the Structured Assessment of Violence Risk in Youth [29], the Psychopathy Check List: Youth Version [30], the Juvenile-Sex Offender Assessment Protocol [31], and the HCR-20 Violence Risk Assessment Scheme [32]. The JFP is related to the adult version of the Forensic Profile list, the FP40 [33]. Both instruments are often used to study the Dutch forensic population. The 70 items are divided into seven domains: ´History of criminal behavior’, ‘Family and environment’, ‘Offense related risk factors and substance abuse’, ‘Psychological factors’, ‘Psychopathology’, ‘Social behavior/interpersonal relationships’ and ‘Behavior during stay in the institution’.

The items are scored on a three point scale with 0 = no problems, 1 = some problems, and 2 = severe problems. Previous studies have demonstrated that the JFP is a solid instrument based on file information, with acceptable inter-rater reliability (r = .73; κ = .61), strong convergent validity with the SAVRY [34], adequate predictive validity [35], adequate face validity and clinical value [36] and overall satisfactory psychometrics qualities [27]. Studies on domain scores across gender in the adult population demonstrated no differences [37].

### Procedure

The JFP-list was scored after 1 year of treatment, since necessary (historical) information is available at that moment and to be able to include (dynamic) risk factors during treatment, such as motivation and attitude towards treatment. All files (n = 2010) were read and scored anonymous with the JFP-list by (psychology or criminology) master-students in their last year before graduation. The students were trained for 3 weeks before scoring the instrument individually. This training included a test of the quality of scoring in order to check the files were read and scored as intended.

### Statistics

During the statistical analyses of this study, sequential steps were made in clustering individuals into subgroups. These steps were based on Everitt [38] and have been previously used in the forensic field [39]. All statistics were calculated with SPSS, IBM, version 24.0.

First, descriptives were calculated. Second, we performed a Principal Axis Factor analysis (PAF) to cluster the 70 items of the JFP-list into dimensions of related items, in order to be able to work with a usable number of variables during cluster-analysis. This reduction in variables was needed as to prevent the effect that is known as the ‘Curse of dimensionality’ [40, 41]. This effect may occur as a large number of variables increases the risk that the variables are less dissimilar and specific aspects covered by these variables can be overrepresented in the clustering solution [42]. Third, cluster analyses were performed. For the present study, a two-step method of cluster analyses was used which starts with hierarchical cluster-analysis, followed by an iterative cluster-analysis to form the subgroups. During hierarchical cluster-analysis 4.5% of 2010 the outliers were removed using the Mahalanobis distance (> 25.0) and Cooke’s distance (> .0050). The information of the hierarchical cluster analysis was used as starting point. In the consecutive steps, the iterative clustering, all case are appointed to a cluster, thus no outliers were removed. Euclidean distance [43, 44], was used together with z-scores ranged 0–1 in order to standardize the distance between subjects. Fourth, Ward’s method, also known as the ‘Minimum sum of squares’ [45], was used to set the distance between clusters, merging at the point that leads to minimum increase in total within-cluster variance. A clinical useful aspect of Ward’s method is that this leads to subgroups with more equal sizes than when other cluster methods are used [46]. Different fit indexes (D-index; Hartigan; Scott; Friedman) were measured for the different cluster solutions. All these measures have an index pointing towards the optimal number of clusters [47, 48]. All cluster solutions are nested, which means that in each consecutive step one cluster is split into two clusters.

Next, we presented the different subgroup-solutions resulting from the cluster-analyses at six clinicians working in JJIs during a group session in order to test the clinical validity of the subgroups. These clinicians were considered experts in their field and included psychiatrist, psychotherapists and psychologists with extensive experience in the treatment of serious juvenile offenders in the JJI or in outpatient settings. Cluster solutions for 5 to 8 clusters were presented, in order to end up with as few clusters as possible to be able to understand them and be practical, but also having enough clusters to identify the subtle differences between clusters [42]. Additional benefit of this step is that the relatively subjective step of choosing the number of clusters is taken away from the researcher [49].

Based on clinical relevance and statistical measures, we choose the optimal cluster solution. During a post hoc comparison with ANOVA’s that focused on the differences between subgroups on factor level and mean item scores the uniqueness of the subgroups were checked.

Finally, we studied the 70 item scores on the different risk factors from the final subgroup solution, in order to find indicators for tailored treatment per subgroup. Post-hoc analyses using ANOVA’s were used, in order to find distinguishing (elevated) item scores between subgroups.

## Results

### Factor analyses

The PAF analyses of the 70 items of the FPJ-list resulted in nine factors, named *Antisocial behavior, Sexual problems, Family background, Mental health problems, Substance use, Conscience and Empathy, Cognitive and social skills, Social network* and *Offenses.* Table 1 demonstrates the 70-items of the JFP-list and the factors they belong to, based on the PAF analyses. Compared to the nine factor solution of Mulder and colleagues, 94.5% out of the 70 items fell under the same factor in this study (see Additional file 1).

**Table 1:** Results of the PAF analyses with items from JFP-list per factor and their loadings

	*N* = *2010*
Factor 1: antisocial behavior during treatment	
Antisocial behavior in institution	.717
Negative coping	.701
Lack of cooperation with treatment	.673
Incidents, aggression in institution	.592
Treatment motivation	.583
Lack of positive coping	.511
Lack of commitment to school/work	.504
Negative attitude in the institution	.415
Lack of contact, trust, openness	–
Factor 2: sexual problems	
Sexual offense	.931
Problematic sexual behavior	.913
Pedosexual behavior	.616
Past offense, searching for a victim	.477
Threat to be involved in prostitution (−)	.381
Involvement in criminal environment (−)	.368
Sadism	.325
Victim of sexual abuse	.316
Truancy (−)	
Factor 3: family background	
Witnessing violence in the family	.647
Lack of consistency of parents/parental control	.605
Presence/accessibility by parents	.584
Problematic family situation	.577
Substance abuse by parents	.552
Criminal behavior of family	.446
Physical/emotional abuse	.445
Psychopathology in parents	.352
Factor 4: mental health problems	
Psychotic symptoms	.542
Offense following psychosis/medication stop	.405
Depression (past year)	.387
Anxiety	.355
Peer rejection	.346
Autism spectrum disorder	.287
Poor selfcare	–
Factor 5: substance use	
Substance use preceding/during the offense	.859
Drugs abuse	.722
Alcohol abuse	.629
Factor 6: conscience and empathy	
Lack of conscience	.618
Lack of empathy	.618
Lack of problem apprehension	.590
Personality traits cluster B	.292
Factor 7: cognitive and social skills	
Low academic achievement	.542
Low IQ	−.469
Low social skills	.361
Self-esteem	.350
Self-reliance	.323
Neurobiological disorder	.249
Suggestibility	–
Previous contact with mental health care services	–
Factor 8: social network	
Network, low quantity	.369
Network, lack of emotional support	.332
Impulse regulation in the past	.316
Cooperative behavior, problems with authorities	.229
ADHD	.219
Coping, avoidance (−)	.218
Lack of social activities	–
Factor 9: offenses	
High number of past offenses	.732
Violent criminal behavior	.501
Young age first conviction	.473
Young age of onset problem behavior	.394

### Cluster-analyses

We used the results of factor analyses as input for the cluster-analyses to identify subgroups with comparable scores over the nine factors. Based on individual scores of 2010 adolescents on these nine factors, cluster-analyses identified four cluster solutions with adequate fit measures, which were presented to clinical experts. Table 2 gives an overview of the four identified subgroups and their fit indexes. Based on these statistics the solution with six clusters, demonstrates the best fit.

**Table 2:** Descriptions of the subgroups from the 5-, 6-, 7- and 8-cluster solutions and their fit measures

	5	6	7	8	Number of optimal clusters
D-index	2.33	2.25	2.23	2.22	6
Hartigan	125.52	35.20	30.92	63.44	6
Scott	2615.53	3478.60	3851.44	4015.40	6
Friedman	2.04	2.81	3.11	3.35	6
Cluster description	Sexual problems	Sexual problems	Sexual problems	Sexual problems	
			Sexual, social and cognitive problems	Sexual, social and cognitive problems	
	Antisocial behavior and multi problems	Antisocial behavior and multi problems	Antisocial behavior and multi problems	Antisocial behavior and multi problems	
		Problems around empathy and conscience	Problems around empathy and conscience	Problems around empathy and conscience	
	Group with mild problems around network	Group with mild problems around network	Group with mild problems around network	Group with mild problems around network	
	Family background problems	Family background problems	Family background problems	Family background problems	
	Substance use problems	Substance use problems	Substance use problems	Substance use problems	
				Substance use and network problems	

The consultation of the clinical experts resulted in a cluster solution of seven subgroups of serious juvenile offenders, since this solution dived the subgroup of juveniles with sexual problems into two subgroups and therefore connected best with clinical practice. The clusters were named after the offender characteristics that differentiated the subgroups from each other: (1) sexual problems, (2) antisocial identity and mental health problems, (3) lack of empathy and conscience, (4) flat profile, (5) family problems, (6) substance use problems, and (7) sexual, cognitive and social problems. Each of the seven subgroups contained between 7 % (n = 141) to 21.1% (n = 424) of the serious juvenile offenders and the females were fairly equally divided over the seven subgroups, with the exception of the sexual problems subgroups (see Additional file 2). The final 7-cluster solution and the mean scores on the factors per cluster are shown in Table 3. The subgroups are listed in order in which the hierarchical cluster-analyses detected the seven subgroups and can be described as follows:

**Table 3:** Mean factor scores per cluster solution (range 0–2) and differences between subgroups on the factors

Nine factor scores	Cluster 1: n = 141	Cluster 2: n = 218	Cluster 3: n = 394	Cluster 4: n = 424	Cluster 5: n = 287	Cluster 6: n = 337	Cluster 7: n = 209	F (df = 6,1894)	Sign.
Antisocial behavior during treatment	− .802	.995	.878	− .628	− .429	− .169	− .013	287.81	*p* < .005
Sexual problems	1.613	− .032	− .393	− .441	− .277	− .451	1.691	823.10	*p* < .005
Family background	− .705	.449	.075	− .549	1.016	− .274	.031	125.43	*p* < .005
Mental health problems	.288	1.177	− .397	− .492	.287	− .316	.440	74.99	*p* < .005
Substance use	− 1.087	.851	− .101	− .413	.094	.986	− .845	249.19	*p* < .005
Conscience and empathy	− .868	.536	.789	− .183	− .606	− .233	.113	177.32	*p* < .005
Cognitive and social skills	− .353	.569	− .073	− .361	− .205	.015	.775	60.72	*p* < .005
Social network	− .240	− .440	− .016	.279	− .237	.372	− .192	29.73	*p* < .005
Offenses	− .586	.035	.280	− .403	− .268	.369	.416	41.19	*p* < .005

Cluster 1: sexual problems, cluster 2: antisocial identity and mental health problems, cluster 3: lack of empathy and conscience, cluster 4: flat profile, 5: family problems, cluster 6: substance use problems, and cluster 7: sexual, cognitive and social problems

#### Subgroup 1: sexual problems

Compared to the other groups, juveniles in this subgroup display predominately problems with sexuality, such as problematic (pedo)sexual behavior or committing a sexual offense. They also display mental health issues such as peer rejection. This subgroup represents 7% of the sample.

#### Subgroup 2: antisocial identity and mental health problems

This group consists of juveniles characterized by antisocial behavior and mental health problems. The prevalence of substance use problems in this subgroup is high, compared to the other subgroups. This subgroup represents 10.8% of the sample.

#### Subgroup 3: lack of empathy and conscience

The juveniles in this subgroup are quite similar to the ones in subgroup 2, but without the mental health and substance use problems. Additionally, these juveniles display a development towards personality disorders in the direction of antisocial, narcissistic of borderline personality disorder. This subgroup represents 19.6% of the sample.

#### Subgroup 4: flat profile

On all domains the scores of these juveniles are relatively average compared to the other subgroups. However, compared to the general population, the problems of these adolescents are still considerable. Juveniles from this profile show the most problems around their social network. This subgroup represents 21.1% of the sample.

#### Subgroup 5: family problems

Compared to the other groups, juveniles in this subgroup mainly experience family problems, such as inconsistent parenting, abuse and witnessing violence in the family. Additionally, these juveniles also have mental health problems as well as substance abuse problems. This subgroup represents 14.3% of the sample.

#### Subgroup 6: substance use problems

These juveniles mainly demonstrate problems with substance abuse, often preceding their offending behavior. They also experience problems in their social network. This subgroup represents 16.8% of the sample.

#### Subgroup 7: sexual, cognitive and social problems

This group consists of juveniles who display problems with sexuality in combination with a lack of social and cognitive skills. Additionally, they display mental health problems. These adolescents have suffered peer rejection and autism spectrum disorders. This subgroup represents 10.4% of the sample.

ANOVA’s resulted in strong significant (*p* < .0005) differences between the seven subgroups on all nine factors, as demonstrated in Table 3. The cluster solution scores from the 2010 study can be found in Additional file 3.

**Fig. 1:**
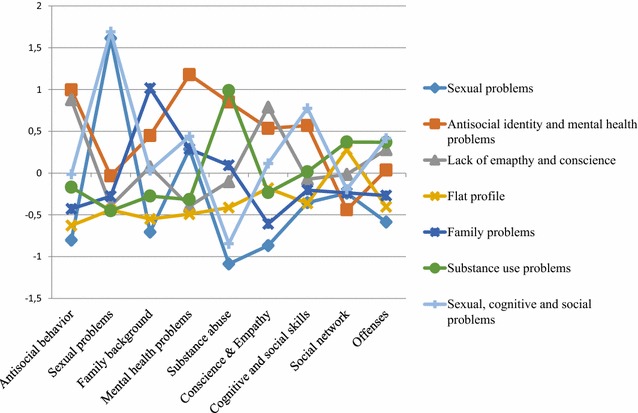
The seven subgroups and their functioning on the different factors

Figure 1 provides a graphical overview of the seven subgroups and their scores on the items belonging to the different factors.

#### Item scores from different subgroups

For each of the nine factors, item scores were compared between the seven subgroups. Since all items are scored on a three point scale (0, 1 or 2), scores in the direction of 2 indicate severe problems. All item scores differed significantly between subgroups (*p* < .0005). The item scores from the different factors and the differences between subgroups will be discussed (see Additional file 4 for numerical values and Additional file 5 for the graphical images).

Regarding the first factor, *Antisocial behavior*, the “Antisocial identity and mental health problems” subgroup (.91–1.31) displayed the highest mean scores on items from this factor, followed by the “Lack of empathy and conscience” subgroup (.91–1.29). These two subgroups had particular high mean scores on the items: ‘negative coping’, ‘lack of positive coping’, and ‘lack of motivation for treatment’. Juveniles from the “Sexual problems” subgroup had the lowest mean scores (.15–.65) on items from this factor.

Turning to the second factor, *Sexual problems,* the highest scores were found in the “Sexual, cognitive and social problems” subgroup (.37–1.75) and the “Sexual problems” subgroup (.15–1.62). High scores were found at the items ‘problematic sexual behavior’, ‘pedosexual behavior’, and ‘sexual offense’. Mean scores on the items ‘threat to be involved in prostitution’ and ‘involvement in criminal environment’ were higher among juveniles from the other five subgroups.

On the third factor, *Family background,* the “Family problems” subgroup (.95–1.77) was the highest scoring subgroup, followed by the “Antisocial identity and mental health problems” subgroup (.66–1.59). These two subgroups displayed the highest scores on the items ‘lack of consistency of parents/parental control’, ‘presence/accessibility of parents’, and ‘physical/emotional abuse’. In contrast, juveniles from the “Sexual problems” subgroup (.17–.82) had the lowest mean scores on the items from this factor.

Regarding the fourth factor, *Mental health problems*, the “Antisocial identity and mental health problems” subgroup had the highest scores in relation to the other subgroups. The items; ‘peer rejection’ (1.04), ‘psychotic symptoms’ (.78), and ‘depression’ (.59) stood out for their high scores. The “Sexual, cognitive and social problems” subgroup and “Sexual problems” subgroup demonstrated the highest scores, compared to the other subgroups, especially for the items ‘peer rejection’ (1.24 and 1.03), and ‘autism spectrum disorder’ (.82 and .66).

Regarding the fifth factor, *Substance abuse*, the “Substance use problems” subgroup (.82–1.55) had the highest scores, followed by the “Antisocial identity and mental health problems” subgroup (.78–1.59). The “Sexual, cognitive and social problems” subgroup (.07–.27) and the “Sexual problems” subgroup (.08–.22) had the lowest scores on items from this factor.

The sixth factor, *Conscience and Empathy,* showed high overall item scores in all seven subgroups. The “Lack of empathy and conscience” subgroup (1.76–1.86) and “Antisocial identity and mental health problems” subgroup (1.61–1.72) had the highest scores on this factor. Items ‘lack of problem apprehension’ and ‘personality traits Cluster B’ demonstrate the highest scores in this factor. The “Sexual problems” subgroup (.80–1.36) had the lowest mean item scores on this factor.

The seventh factor, *Cognitive and social skills,* demonstrated the highest scores for the “Sexual, cognitive and social problems” subgroup (.47–1.58) and “Antisocial identity and mental health problems” subgroup (.26–1.36). All subgroups had the highest mean item score on the item ‘self-esteem’.

Regarding the eighth factor, mean item scores on factor *Social network* were highest in the “Antisocial identity and mental health problems” subgroup (.63–1.69), especially on the item ‘cooperative behavior, problems with authority’. The “Sexual problems” subgroup had the lowest item scores on this factor (.28–.91).

Finally, item scores on the ninth factor *Offenses* demonstrated quite similar scores for all subgroups. The “Substance use” subgroup (.40–1.64) and “Lack of empathy and conscience” subgroup had the relatively highest items scores on these factor (.34–1.61).

## Discussion

Present study aimed to identify subgroups of serious juvenile offenders in JJIs based on specific sets of offender characteristics that can serve as an important starting point for tailored treatment. Cluster-analyses in a sample of 2010 serious juvenile offenders and checks of cluster solutions by clinicians resulted in seven subgroups of serious juvenile offenders: (1) a sexual problems subgroup, (2) an antisocial identity and mental health problems subgroup (3) a lack of empathy and conscience subgroup, (4) a flat profile subgroup, (5) a family problems subgroup, (6) a substance use problems subgroup, and (7) a sexual, cognitive and social problems subgroup.

The present study is a replication of the previous study by Mulder and colleagues [23] and thereby a validation of the earlier described subgroups. Factor analyses on the 70 items of the JFP-list of risk factors demonstrated almost the same nine factors in the present as the previous study on a sample twice as large. This implies that the risk factors of the JFP-list are consistently divided over nine factors. Results of the present study further indicate towards a good replication of the identification of robust subgroups of serious juvenile offenders. The original study identified six subgroups, whereas the present study identified seven subgroups. The present six subgroups were supplemented with a subgroup of juveniles marked by substance use.

Although the other subgroups are more or less identical between the studies of 2010 and 2017, the subgroup of offenders with substance use problems is remarkable. This especially because it was not the last cluster that originated from a larger subgroup during the hierarchical cluster-analyses, which could imply that it is a subgroup of a subgroup. Furthermore, this subgroup is fairly large (16.8%). These results suggest that over the years problematic alcohol and drugs use in Dutch serious juvenile offenders increased to the extent that it influences delinquent behavior. Statistics from the Trimbos Instituut, the Dutch institute for mental health and substance use, show, however, a decrease in the use of alcohol, soft-drugs and hard-drugs since 2003 in the total population of Dutch adolescents [50]. Research that focused specifically on the population in the JJI in the Netherlands has shown different substance use behavior, since the problematic use of alcohol and the use of substances during criminal behavior has increased between the years 1995 and 2010 [51]. The specific subgroup of serious juvenile offenders with substance use problems was also acknowledged by the clinicians as a separate group in their existence and need for a specific approach during treatment, which will be discussed hereafter.

Including female adolescents and adolescents from a large age-span in the current sample, provided the opportunity to find out whether these groups form a separate subgroup based on their offender characteristics. This was not the case, since the female, younger and older adolescents were distributed over the subgroups that were found. However, the present sample includes only a small percentage of females or older adolescents and therefore can present findings not be generalized to these groups of serious juvenile offenders. Thereby, prevalent theories on the development of criminal behavior [14, 52] discuss the differences between boys and girls that are also seen in clinical practice. Further research into these subgroups, with larger samples is needed in order to be able to say anything conclusive about female or older serious juvenile offenders.

Since the present study is based on a large sample and identified almost the same subgroups as the previous study, based on solid performed cluster-analyses that are also validated with clinical experts, we feel confident to adopt these seven subgroups and take a closer look at the characteristics of the juveniles per subgroup. It is not possible to develop a set protocol for the treatment of serious juvenile offenders per subgroup. However, insight can be given in treatment ingredients towards specific offender characteristics and tailored treatment. The interventions suggested are not new, but they can be seen as suggestions to support clinicians tailoring treatment depending on subgroup characteristics and in this way focus on the most important factors for the specific individual in treatment. Next, the unique offender characteristics per subgroup will be described to be able to point towards treatment indicators per subgroup, but not before we pay attention to the following issue. Although present subgroups are the result of extended analyses in a large sample of serious juvenile offenders and of high clinical relevance, it is important to interpret current results with some caution. As always when making classifications, different nuances can be found in offender characteristics. Not all serious juvenile offenders who cluster into a subgroup are exactly the same, although they do share distinguishing characteristics that could be relevant for treatment and treatment outcome. Since young offenders are still developing, subgroups also need to be put in a developmental perspective.

A large part of the serious juvenile offenders belong to the “Lack of empathy and conscience” subgroup. As this profile is quite common, all clinical practitioners in juvenile justice institutions should be equipped with adequate intervention techniques to promote the development of empathy and conscience. This is in line with clinical practice as most correctional programs are working on increasing empathy. However, studies on the effect of these interventions are still scarce. Specific interventions based on cognitive behavioral therapy (CBT) that work on critical and moral reasoning, social skills and empathy have shown promising results [53] and could therefore be suitable for treatment of offenders from this subgroup. This subgroup further displays high risks to develop personality disorders (antisocial, borderline, narcissistic), negative coping styles and orientation towards a criminal environment. Suitable interventions to work on these criminogenic factors are based on CBT and schema focused therapy with training in social skills and problem solving or focus in the system of the juvenile like Multi Systemic Therapy (MST) [54].

Another subgroup identified in this study was the subgroup “Substance use problems”. This indicates that it is essential to pay special attention to these problems, all the more because previous studies have demonstrated high prevalence rates (60%) of substance use problems in detained adolescents [2, 55, 56]. It has been stated that substance abuse and delinquent behavior could have a different etiology and are linked with different psychological and social processes. Interventions focused on reducing substance abuse and those focused on reducing reoffending behavior should therefore aim at different processes and mechanisms [57]. Promising interventions for substance abuse problems for serious juvenile offenders are cognitive behavioral therapy and Multi-Dimensional Family therapy (MDFT) [58].

The subgroup “Antisocial identity and mental health problems” contains serious juvenile offenders with a clear antisocial identity. Negative coping style, lack of motivation for treatment and a negative attitude have a particular high prevalence in these juveniles. Although it is important for every juvenile from every subgroup to address motivation, juveniles from the subgroup “Antisocial identity” seem to have specific problems concerning their attitude towards treatment and motivation for change, compared to other subgroups. Therefore it seems important to focus on motivating and engaging with the adolescent first, in order to be able to work on underlying problems at a later stage. In order to develop motivation for treatment, the juvenile needs to be provided with the optimal balance of autonomy, competence and relatedness [59]. Motivation for treatment is found to be a crucial factor for engaging juveniles in the process of change in treatment trajectories [60] and reducing the risk of reoffending [61].

Further, the results reveal two separate subgroups of offenders that demonstrate sexual problems (sexual problems and sexual, cognitive and social problems). The presence of committing a sexual offense, however, does not mean that all these juveniles are identified as a member of one of these two subgroups. Around a third of the sexual offenders from the current sample (167 from 467) was divided over the other five subgroups, with the largest part in the “Lack of empathy and conscience” subgroup. This is in line with previous studies that describe differences within the group of juvenile sex offenders and suggest differentiation in treatment approaches [17, 18, 62]. According to the clinicians participating in a discussion group with focus on the different subgroup solutions, the juveniles with cognitive, social and sexual problems need a different approach than the juveniles only displaying sexual problems, since the first group is more vulnerable and has different needs with respect to reintegration. It has been stated that the (sexual deviant) behavior of these juvenile is more visible and less sophisticated [60] and therefore needs more practical corrections, whereas the other group of sexual offenders has developmental needs on more cognitive and moral level. Further investigation of the differences in item scores between sexual offenders that are included in the different subgroups could provide data driven starting points for treatment.

Further, a distinct subgroup was identified as the “Family problem” subgroup, including lack of consistency in parenting, presence/accessibility of parents and criminal behavior of the parent. Although these are static risk factors and might not be present at the time of incarceration, they may still influence family interactions. It could be important to identify juveniles with these specific characteristics and start family oriented intervention at an early stage of treatment. For example, MST [63], Functional Family Therapy (FFT) [64] and MDFT [65] have shown promising results on family factors as well as other offender characteristics [66, 67].

A strength of this study is that results lead to subgroups with specific characteristics that are of great practical value when creating tailored treatment in JJIs. Clinicians know, based on these results, which  offender characteristics are distinguishing between the population of serious juvenile offenders, need focus during treatment and might point towards missing information that is necessary to develop a suitable treatment trajectory. The use of fit values for the cluster-solutions in combination with the face value of the clinicians, strengthens our findings and overcomes limitations of the identification of subgroups in other studies when the choice of the optimal subgroup solution is often made by the researcher [49]. The large sample on which this study is performed makes the results relevant for a large population of serious juvenile offenders. Thereby, the identification of seven subgroups with distinguishing offender characteristics makes it possible to perform future research on the effects of treatment interventions for different groups of serious juvenile offenders. Currently, evaluation studies of treatment interventions in incarcerated adolescents use a relatively heterogeneous population, while the evaluation of more homogeneous groups as presented in the subgroups in this study could reveal a more realistic outcome of treatment. This makes it possible to not only study *what works?*, but also *what works for whom?* The next step in research should be focused on the experience of the clinician working with juveniles from these different clusters to gain information about best practice interventions, since the identification of the subgroups was data-driven and not theory-driven. This information should be transferred to the clinical field in order to be of great value for clinicians as well as the juveniles. Together with information on future delinquent behavior of juveniles from the seven subgroups this provides practical information on the characteristics that could be targeted to maximize treatment effect in each subgroup.

Notwithstanding the strengths of this study, some limitations must be mentioned. The present study focused on file information of characteristics of Dutch serious juvenile offenders placed in JJIs and therefore focused on a specific group of young offenders. The fact that file based information is used may have led to missed information that was not present in the files, for instance on protective factors or trauma. Moreover, the list that is used to collect the data focused on risk factors and, thereby, overlooks the value of protective elements in a juveniles life. Thereby, the interrater reliability and other psychometric characteristics of the JFP-list for the total current sample were not measured, only for a part of the sample. Present results and the use of the JFP-list to identify subgroups of serious juvenile offender would be stronger if these measures of the total sample could be provided and is the focus of future research. The strength of the large sample in current study can also be regarded as a limitation, because the sample includes a small percentage of girls (4.9%), younger (< 14 years, 4.6%) and older adolescents (> 20 years 3.5%). We were interested in the total population of serious juvenile offenders with a mandatory treatment order and therefore included all juveniles in the sample. When preferring a more homogeneous set of data, these theoretical outliers that appear in daily practice, should be excluded. Present study can be considered an exploration of serious juvenile offenders, as the sample consists of the total population of serious juvenile offenders under a mandatory treatment order in Dutch JJI’s, where also some female offenders and older juveniles reside. Future research based on these specific ‘subgroups’ of serious juvenile offenders is necessary in order to be able to generalize current result to female, younger or older serious juvenile offenders. The results of present study are based on risk factors and offender characteristics of serious juvenile offenders that can be measured in other countries as well and are known factors in international literature. Therefore, the main focus of current results are internationally generalizable: The group of serious juvenile offenders is heterogeneous and there are specific groups (with sexual problems, substance use problems, family problems, antisocial behavior, conscience and empathy problems) with specific needs. Nevertheless, in most Western countries are serious juvenile offenders placed in different facilities and the population of juveniles in JJI’s differs across countries. Future research is necessary to be able to study the international generalizability of our results.

## Conclusions

Present study identified seven subgroups of serious juvenile offenders with distinguishing offender characteristics. Because these subgroups all have their own specific combination of offender characteristics and risk factors, they provide information for clinical practice to apply this knowledge to daily practice and tailored treatment to the needs and possibilities of each specific subgroup. Clinicians should identify specific offender characteristics in order to find suitable intervention strategies for the individual juvenile in the JJI.

## Additional files

**Additional file 1.** Results of the PAF analyses with items from JFP-list per factor and their loadings from 2017 and 2010.

**Additional file 2.** Division of female, younger and older serious juvenile offenders over the total sample (N = 2010).

**Additional file 3.** Mean factor scores per cluster solution (range 0–2) on the factors in 2010.

**Additional file 4.** Mean scores on factor items per subgroups (range 0–2) and the differences between subgroups in 2017.

**Additional file 5.** Item functioning of the seven subgroups on the factor Antisocial behavior. Item functioning of the seven subgroups on the factor Sexual problems. Item functioning of the seven subgroups on the factor Family background. Item functioning of the seven subgroups on the factor Mental health problems. Item functioning of the seven subgroups on the factor Substance use. Item functioning of the seven subgroups on the factor Conscience and empathy. Item functioning of the seven subgroups on the factor Cognitive and social skills. Item functioning of the seven subgroups on the factor Social network. Item functioning of the seven subgroups on the factor Offenses.
