# Supplementary material for: Serious juvenile offenders: classification into subgroups based on static and dynamic charateristics
Source: Child Adolesc Psychiatry Ment Health. 2017 Dec 22;11:67. doi: 10.1186/s13034-017-0201-4 (PMC5740506; doi:10.1186/s13034-017-0201-4)
Supplement: Supplementary file 1 — Additional file 1. Results of the PAF analyses with items from JFP-list per factor and their loadings from 2017 and 2010. [file 13034_2017_201_MOESM1_ESM.docx]

**Additional file 1**

*Results of the PAF analyses with items from FPJ-list per factor and their loadings from 2017 and 2010.*

|  | *2017*  *N=2010* | *2010*  *N=1107* |
| --- | --- | --- |
| **Factor 1 Antisocial behavior during treatment** |  |  |
| Antisocial behavior in institution | .717 | .704 |
| Negative coping | .701 | .669 |
| Lack of cooperation with treatment | .673 | .640 |
| Incidents, aggression in institution | .592 | .551 |
| Treatment motivation | .583 | .536 |
| Lack of positive coping | .511 | .444 |
| Lack of commitment to school/work | .504 | .455 |
| Negative attitude in the institution | .415 | .540 |
| Lack of contact, trust, openness | - | .346 |
| **Factor 2: Sexual problems** |  |  |
| Sexual offense | .931 | .710 |
| Problematic sexual behavior | .913 | .858 |
| Pedosexual behavior | .616 | .654 |
| Past offense, searching for a victim | .477 | .498 |
| Threat to be involved in prostitution (-) | .381 | - |
| Involvement in criminal environment (-) | .368 | .415 |
| Sadism | .325 | .338 |
| Victim of sexual abuse | .316 | .387 |
| Truancy (-) |  | .256 |
| **Factor 3: Family background** |  |  |
| Witnessing violence in the family | .647 | .660 |
| Lack of consistency of parents/parental control | .605 | .622 |
| Presence/accessibility by parents | .584 | .521 |
| Problematic family situation | .577 | .439 |
| Substance abuse by parents | .552 | .577 |
| Criminal behavior of family | .446 | .427 |
| Physical/emotional abuse | .445 | .437 |
| Psychopathology in parents | .352 | .352 |
| **Factor 4: Mental health problems** |  |  |
| Psychotic symptoms | .542 | .660 |
| Offense following psychosis/medication stop | .405 | .547 |
| Depression (past year) | .387 | .312 |
| Anxiety | .355 | .247 |
| Peer rejection | .346 | .283a |
| Autism spectrum disorder | .287 | .299a |
| Poor selfcare | - | .303 |
| **Factor 5: Substance use** |  |  |
| Substance use preceding/during the offense | .859 | .806 |
| Drugs abuse | .722 | .688 |
| Alcohol abuse | .629 | .604 |
| **Factor 6: Conscience and empathy** |  |  |
| Lack of conscience | .618 | .602 |
| Lack of empathy | .618 | .658 |
| Lack of problem apprehension | .590 | .453 |
| Personality traits Cluster B | .292 | .402 |
| **Factor 7: Cognitive and social skills** |  |  |
| Low academic achievement | .542 | .487 |
| Low IQ | -.469 | -.294 |
| Low social skills | .361 | .420 |
| Self-esteem | .350 | - |
| Self-reliance | .323 | - |
| Neurobiological disorder | .249 | - |
| Suggestibility | - | .329 |
| Previous contact with mental health care services | - | .273 |
| **Factor 8: Social network** |  |  |
| Network, low quantity | .369 | .496 |
| Network, lack of emotional support | .332 | .431 |
| Impulse regulation in the past | .316 | - |
| Cooperative behavior, problems with authorities | .229 | - |
| ADHD | .219 | .286b |
| Coping, avoidance (-) | .218 | - |
| Lack of social activities | - | .336 |
| **Factor 9: Offenses** |  |  |
| High number of past offenses | .732 | .791 |
| Violent criminal behavior | .501 | -.424 |
| Young age first conviction | .473 | .559 |
| Young age of onset problem behavior | .394 | .313b |

*a In 2010 factor: Sexual problems*

*b In 2010 factor: Cognitive and social skills*

*-= reversed scores 0=2, 2=0*
