# Supplementary material for: Serious juvenile offenders: classification into subgroups based on static and dynamic charateristics
Source: Child Adolesc Psychiatry Ment Health. 2017 Dec 22;11:67. doi: 10.1186/s13034-017-0201-4 (PMC5740506; doi:10.1186/s13034-017-0201-4)
Supplement: Supplementary file 2 — Additional file 2. Division of female, younger and older serious juvenile offenders over the total sample (N = 2010). [file 13034_2017_201_MOESM2_ESM.docx]

**Additional file 2**

*Division of female, younger and older serious juvenile offenders over the total sample (N=2010).*

|  | Subgroup 1  N (%) | Subgroup 2 | Subgroup 3 | Subgroup 4 | Subgroup 5 | Subgroup 6 | Subgroup 7 |
| --- | --- | --- | --- | --- | --- | --- | --- |
| Male | 168 (99.4) | 246 (95.0) | 461 (94.3) | 296 (94.0) | 137 (89.0) | 338 (95.2) | 151 (98.1) |
| Female | 1 (0.6) | 13 (5.0) | 28 (5.7) | 19 (6.0) | 17 (11.0) | 17 (4.8) | 3 (1.9) |
| < 15 years old | 22 (13.0) | 23 (8.9) | 32 (6.5) | 8 (2.5) | 4 (2.6) | 10 (2.8) | 17 (11) |
| 15-21 years old  > 22 years old | 147 (87)  0 (0) | 235 (90.7)  1 (0.4) | 457 (93.5)  0 (0) | 307 (97.5)  0 (0) | 150 (97.4)  0 (0) | 344 (96.9)  1 (0.3) | 136 (88.3)  1 (0.6) |

*Subgroup 1) sexual problems, Subgroup 2) antisocial identity and mental health problems, Subgroup 3) lack of empathy and conscience, Subgroup 4) flat profile, 5) family problems, Subgroup 6) substance use problems, and Subgroup 7) sexual, cognitive and social problem*
