# Supplementary material for: Serious juvenile offenders: classification into subgroups based on static and dynamic charateristics
Source: Child Adolesc Psychiatry Ment Health. 2017 Dec 22;11:67. doi: 10.1186/s13034-017-0201-4 (PMC5740506; doi:10.1186/s13034-017-0201-4)
Supplement: Supplementary file 3 — Additional file 3. Mean factor scores per cluster solution (range 0–2) on the factors in 2010. [file 13034_2017_201_MOESM3_ESM.docx]

**Additional file 3**

*Mean factor scores per cluster solution (range 0-2) on the factors in 2010.*

| **Nine factor scores 2010** | **Cluster 1: n=233** | **Cluster 2: n=128** | **Cluster 3: n=336** | **Cluster 4: n=113** | **Cluster 5: n=86** | **Cluster 6: n=211** |
| --- | --- | --- | --- | --- | --- | --- |
| Antisocial behavior during treatment | 1.052 | .195 | -.370 | .163 | -.595 | -.542 |
| Sexual problems | -.194 | -.481 | -.470 | 1.592 | 1.405 | -.319 |
| Family background | .401 | -.197 | -.519 | .011 | -.610 | .779 |
| Mental health problems | .380 | -.459 | -.438 | .303 | .261 | .025 |
| Substance use | .532 | .584 | -.062 | -.753 | -1.049 | -.016 |
| Conscience and empathy | .601 | .221 | .126 | .199 | -.980 | -.685 |
| Cognitive and social skills | .454 | .-175 | -.368 | .721 | -.543 | .085 |
| Social network | .348 | .135 | -.282 | .217 | -.301 | -.027 |
| Offenses | -.024 | 1.260 | -.318 | -.135 | -.477 | -.149 |

*Cluster 1) antisocial identity, Cluster 2) frequent offenders, Cluster 3) flat profile, Cluster 4) sexual problems and weak social identity, Cluster 5) sexual problems, and Cluster 6) problematic family background.*
