# Supplementary material for: Serious juvenile offenders: classification into subgroups based on static and dynamic charateristics
Source: Child Adolesc Psychiatry Ment Health. 2017 Dec 22;11:67. doi: 10.1186/s13034-017-0201-4 (PMC5740506; doi:10.1186/s13034-017-0201-4)
Supplement: Supplementary file 4 — Additional file 4. Mean scores on factor items per subgroups (range 0–2) and the differences between subgroups in 2017. [file 13034_2017_201_MOESM4_ESM.docx]

**Additional file 4**

*Mean scores on factor items per subgroups (range 0-2) and the differences between subgroups in 2017.*

|  | **Cluster 1** | | **Cluster 2** | **Cluster 3** | **Cluster 4** | **Cluster 5** | **Cluster 6** | **Cluster 7** | ***F*** | ***Sig.*** |
| --- | --- | --- | --- | --- | --- | --- | --- | --- | --- | --- |
| **Factor 1 Antisocial behavior during treatment** |  | |  |  |  |  |  |  |  |  |
| Antisocial behavior in institution | .34 | | 1.08 | 1.14 | .35 | .42 | .57 | .65 | 99.92 | *p* < 0.005 |
| Negative coping | .54 | | 1.31 | 1.29 | .61 | .63 | .88 | .94 | 76.98 | *p* < 0.005 |
| Lack of cooperation with treatment | .24 | | .91 | .93 | .20 | .31 | .43 | .43 | 103.85 | *p* < 0.005 |
| Incidents, aggression in institution | .15 | | .92 | .91 | .24 | .31 | .51 | .49 | 50.68 | *p* < 0.005 |
| Treatment motivation | .29 | | 1.11 | 1.13 | .35 | .42 | .37 | .58 | 98.19 | *p* < 0.005 |
| Lack of positive coping | .65 | | 1.28 | 1.20 | .64 | .81 | .79 | 1.09 | 54.84 | *p* < 0.005 |
| Lack of commitment to school/work | .34 | | 1.01 | .78 | .34 | .47 | .57 | .62 | 42.32 | *p* < 0.005 |
| Negative attitude in the institution | .36 | | 1.14 | 1.01 | .34 | .61 | .43 | .50 | 71.83 | *p* < 0.005 |
| **Factor 2: Sexual problems** | |  |  |  |  |  |  |  |  |  |
| Sexual offense | | .87 | .14 | .17 | .07 | .09 | .10 | .96 | 307.10 | *p* < 0.005 |
| Problematic sexual behavior | | 1.62 | .48 | .30 | .12 | .16 | .21 | 1.75 | 320.49 | *p* < 0.005 |
| Pedosexual behavior | | .90 | .03 | .01 | .01 | .02 | .02 | 1.04 | 246.80 | *p* < 0.005 |
| Past offense, searching for a victim | | .42 | .07 | .04 | .00 | .01 | .02 | .42 | 51.11 | *p* < 0.005 |
| Threat to be involved in prostitution (-) | | .21 | 1.12 | 1.18 | .90 | .74 | 1.26 | .27 | 44.72 | *p* < 0.005 |
| Involvement in criminal environment (-) | | .15 | 1.14 | 1.50 | 1.18 | .95 | 1.48 | .41 | 148.09 | *p* < 0.005 |
| Sadism | | .16 | .19 | .11 | .02 | .03 | .06 | .37 | 27.33 | *p* < 0.005 |
| Victim of sexual abuse | | .45 | .32 | .03 | .06 | .29 | .09 | .69 | 48.43 | *p* < 0.005 |
| **Factor 3: Family background** |  | |  |  |  |  |  |  |  |  |
| Witnessing violence in the family | .35 | | .84 | .62 | .23 | 1.36 | .39 | .71 | 65.71 | *p* < 0.005 |
| Lack of consistency of parents/parental control | .82 | | 1.59 | 1.51 | 1.11 | 1.77 | 1.27 | 1.35 | 64.66 | *p* < 0.005 |
| Presence/accessibility by parents | .64 | | 1.26 | 1.10 | .74 | 1.44 | .83 | .10 | 50.74 | *p* < 0.005 |
| Problematic family situation | .24 | | .79 | .63 | .26 | 1.19 | .39 | .55 | 62.06 | *p* < 0.005 |
| Substance abuse by parents | .28 | | .72 | .38 | .21 | 1.10 | .42 | .50 | 45.23 | *p* < 0.005 |
| Criminal behavior of family | .17 | | .66 | .88 | .63 | .95 | .62 | .49 | 17.24 | *p* < 0.005 |
| Physical/emotional abuse | .52 | | 1.05 | .71 | .40 | 1.35 | .55 | .97 | 48.85 | *p* < 0.005 |
| **Factor 4: Mental health problems** |  | |  |  |  |  |  |  |  |  |
| Psychotic symptoms | .11 | | .78 | .03 | .06 | .18 | .10 | .16 | 83.93 | *p* < 0.005 |
| Offense following psychosis/medication stop | .03 | | .26 | .00 | .00 | .02 | .02 | .01 | 50.38 | *p* < 0.005 |
| Depression (past year) | .30 | | .59 | .14 | .15 | .48 | .32 | .31 | 27.57 | *p* < 0.005 |
| Anxiety | .30 | | .51 | .11 | .09 | .36 | .08 | .28 | 31.68 | *p* < 0.005 |
| Peer rejection | 1.03 | | 1.04 | .40 | .30 | .57 | .35 | 1.24 | 76.91 | *p* < 0.005 |
| Autism spectrum disorder | .66 | | .40 | .07 | .09 | .12 | .10 | .82 | 72.36 | *p* < 0.005 |
| **Factor 5: Substance use** |  | |  |  |  |  |  |  |  |  |
| Substance use preceding/during the offense | .08 | | .92 | .24 | .18 | .53 | 1.17 | .07 | 182.38 | *p* < 0.005 |
| Drugs abuse | .22 | | 1.59 | .73 | .60 | 1.15 | 1.55 | .27 | 193.83 | *p* < 0.005 |
| Alcohol abuse | .17 | | .78 | .23 | .25 | .37 | .82 | .10 | 60.37 | *p* < 0.005 |
| **Factor 6: Conscience and empathy** |  | |  |  |  |  |  |  |  |  |
| Lack of conscience | 1.13 | | 1.66 | 1.76 | 1.44 | 1.23 | 1.37 | 1.53 | 54.34 | *p* < 0.005 |
| Lack of empathy | 1.36 | | 1.68 | 1.80 | 1.45 | 1.17 | 1.45 | 1.67 | 52.09 | *p* < 0.005 |
| Lack of problem apprehension | 1.25 | | 1.72 | 1.86 | 1.51 | 1.19 | 1.35 | 1.62 | 67.82 | *p* < 0.005 |
| Personality traits Cluster B | .80 | | 1.61 | 1.81 | 1.54 | 1.39 | 1.67 | 1.21 | 60.09 | *p* < 0.005 |
| **Factor 7: Cognitive and social skills** |  | |  |  |  |  |  |  |  |  |
| Low academic achievement | .53 | | .87 | .62 | .47 | .48 | .57 | 1.04 | 23.38 | *p* < 0.005 |
| Low IQ | .20 | | .26 | .24 | .23 | .19 | .21 | .38 | 8.68 | *p* < 0.005 |
| Low social skills | .74 | | 1.39 | .80 | .33 | .62 | .41 | 1.31 | 122.57 | *p* < 0.005 |
| Self-esteem | 1.37 | | 1.63 | 1.26 | 1.35 | 1.36 | 1.5 | 1.58 | 10.96 | *p* < 0.005 |
| Self-reliance | .26 | | .78 | .30 | .11 | .23 | .16 | .60 | 60.08 | *p* < 0.005 |
| Neurobiological disorder | .21 | | .33 | .12 | .07 | .20 | .24 | .47 | 22.44 | *p* < 0.005 |
| **Factor 8: Social network** |  | |  |  |  |  |  |  |  |  |
| Network, low quantity | .91 | | 1.56 | 1.36 | .97 | 1.33 | 1.11 | 1.32 | 38.03 | *p* < 0.005 |
| Network, lack of emotional support | .39 | | 1.00 | .80 | .38 | .84 | .42 | .70 | 36.09 | *p* < 0.005 |
| Impulse regulation in the past | .86 | | 1.69 | 1.63 | 1.23 | 1.18 | 1.52 | 1.4 | 43.97 | *p* < 0.005 |
| Cooperative behavior, problems with authorities | .44 | | 1.65 | 1.79 | 1.28 | 1.15 | 1.69 | 1.19 | 98.08 | *p* < 0.005 |
| ADHD | .28 | | .63 | .38 | .3 | .22 | .68 | .84 | 28.38 | *p* < 0.005 |
| Coping, avoidance (-) | .81 | | .94 | .51 | .51 | .82 | .52 | .91 | 22.93 | *p* < 0.005 |
| **Factor 9: Offenses** |  | |  |  |  |  |  |  |  |  |
| High number of past offenses | .21 | | .26 | .34 | .23 | .23 | .40 | .31 | 21.98 | *p* < 0.005 |
| Violent criminal behavior | .63 | | .70 | .79 | .68 | .68 | .78 | .79 | 16.14 | *p* < 0.005 |
| Young age first conviction | .93 | | 1.57 | 1.61 | 1.28 | 1.37 | 1.64 | 1.73 | 48.81 | *p* < 0.005 |

*Cluster 1) sexual problems, Cluster 2) antisocial identity and mental health problems, Cluster 3) lack of empathy and conscience, Cluster 4) flat profile, Cluster 5) family problems, Cluster 6) substance use problems, and Cluster 7) sexual, cognitive and social problems*
