# Supplementary material for: Serious juvenile offenders: classification into subgroups based on static and dynamic charateristics
Source: Child Adolesc Psychiatry Ment Health. 2017 Dec 22;11:67. doi: 10.1186/s13034-017-0201-4 (PMC5740506; doi:10.1186/s13034-017-0201-4)

**Additional file 5**

*Item functioning of the seven subgroups on the factor Antisocial behavior*


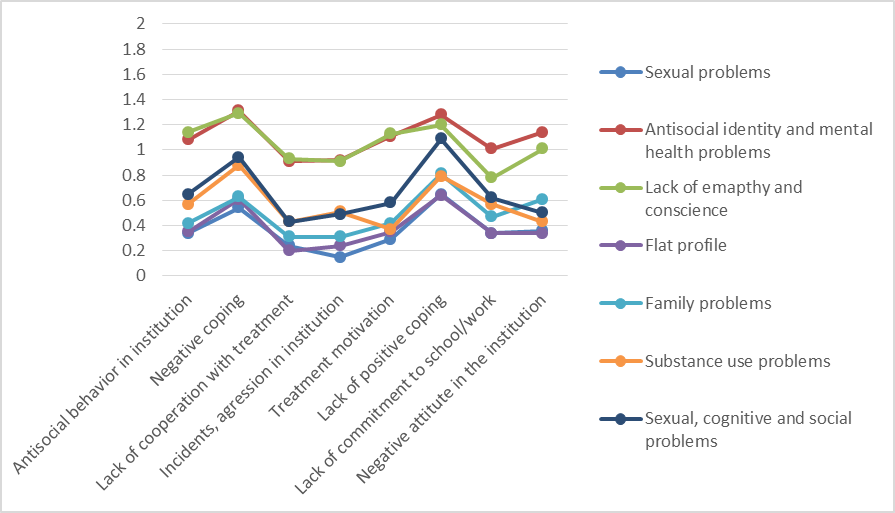


*Item functioning of the seven subgroups on the factor Sexual problems*


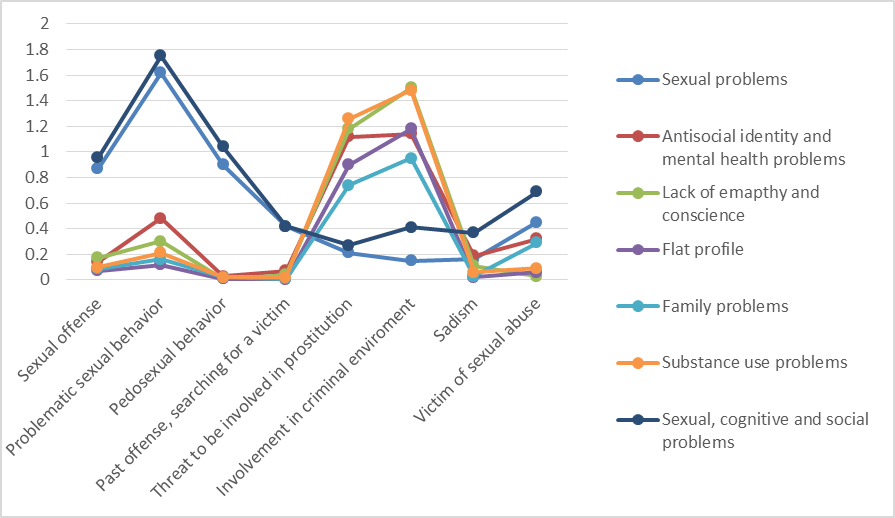


*Item functioning of the seven subgroups on the factor Family background*


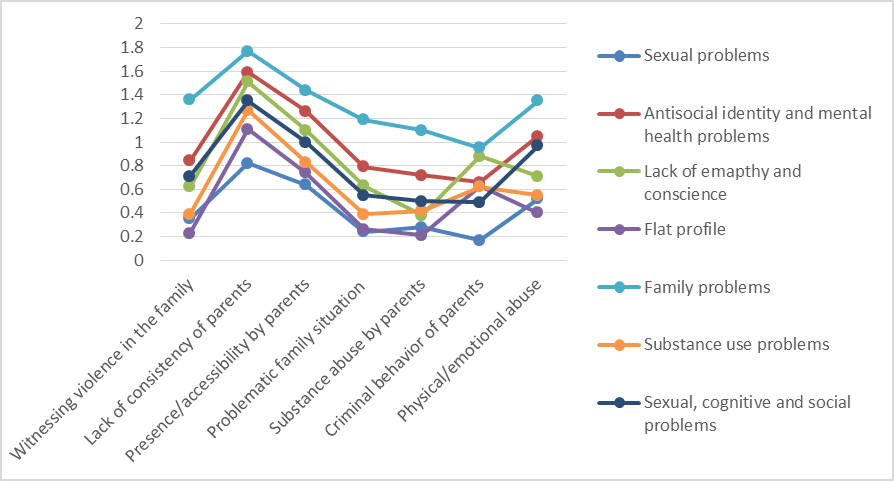


*Item functioning of the seven subgroups on the factor Mental health problems*


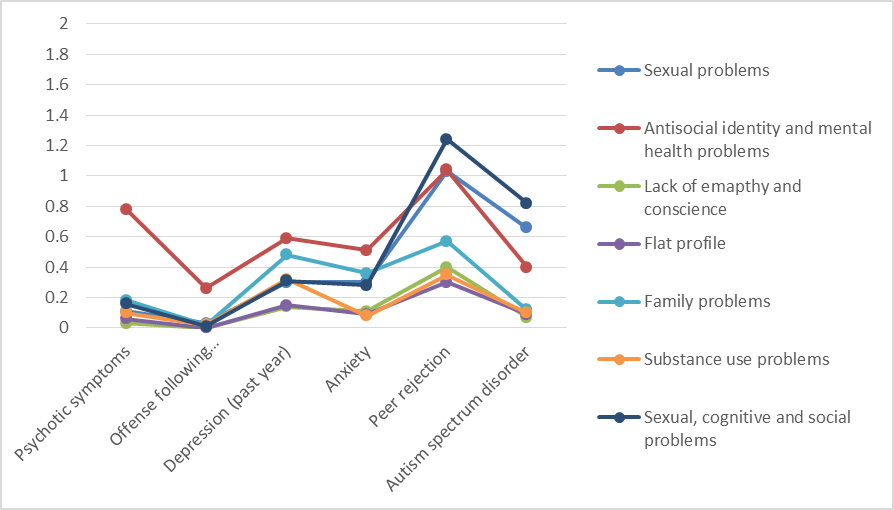


*Item functioning of the seven subgroups on the factor Substance use*


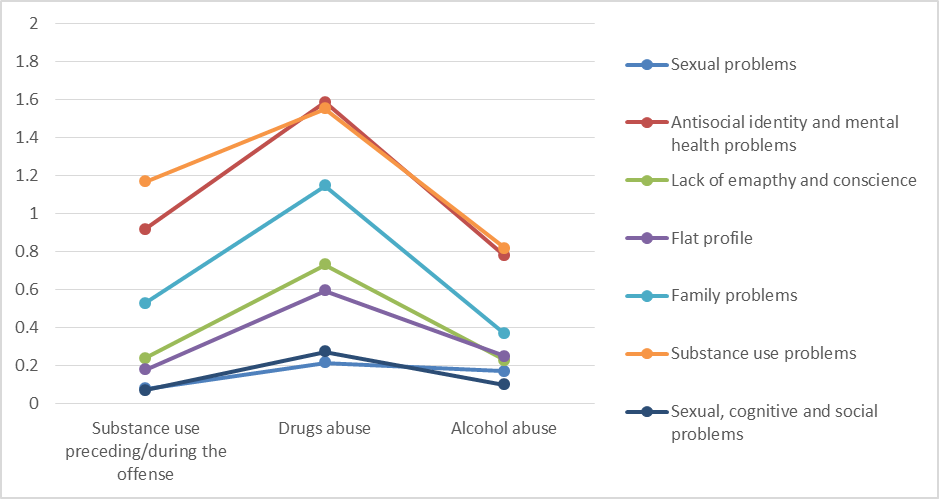


*Figure 7: Item functioning of the seven subgroups on the factor Conscience and empathy*


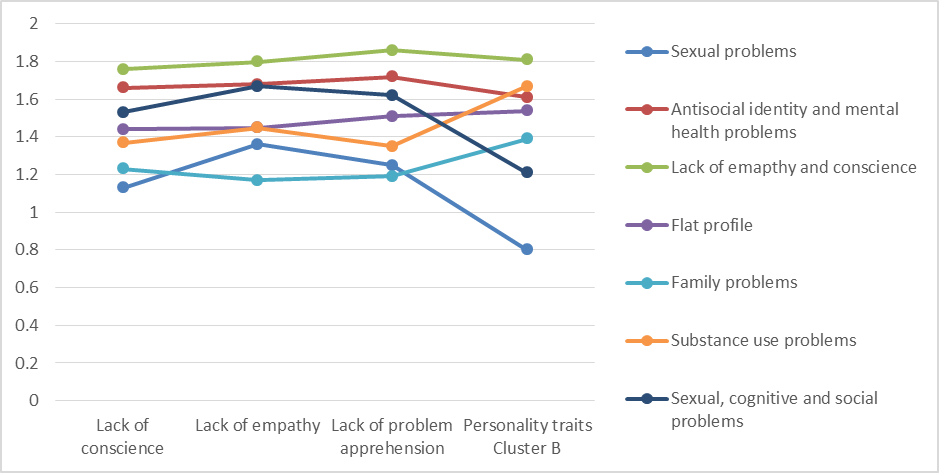


*Item functioning of the seven subgroups on the factor Cognitive and social skills*


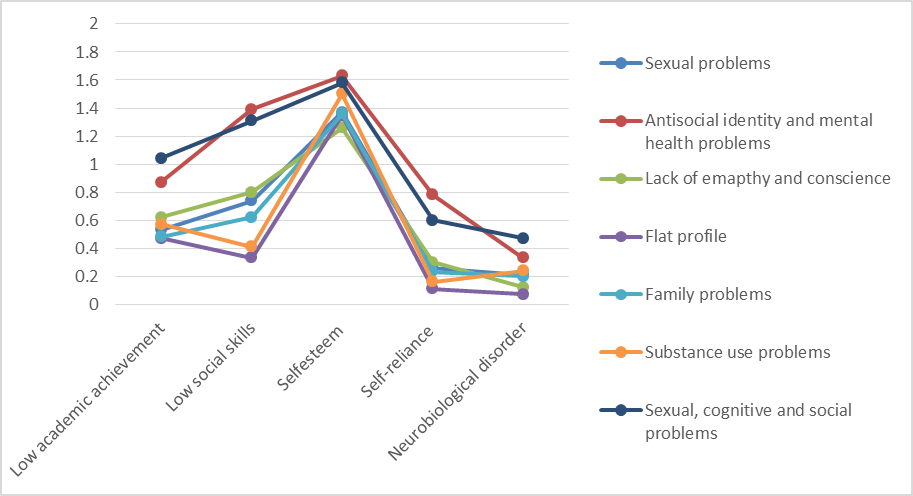


*Item functioning of the seven subgroups on the factor Social network*


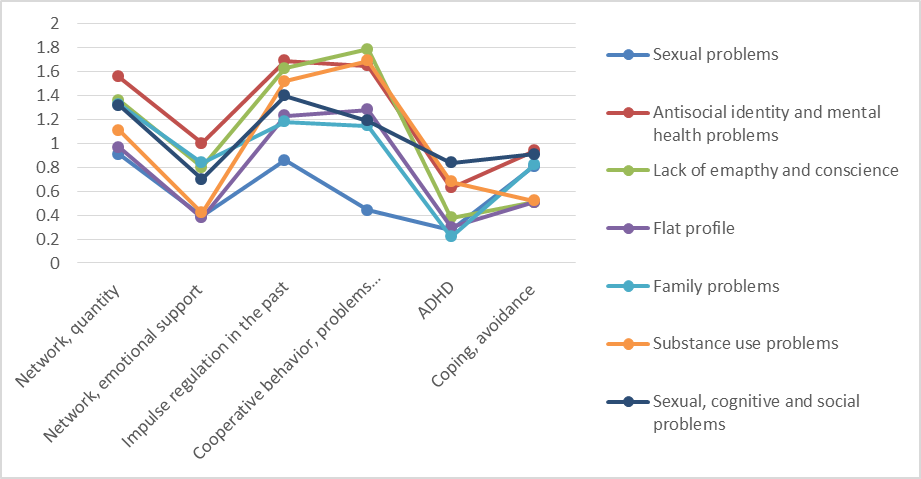


*Item functioning of the seven subgroups on the factor Offenses*


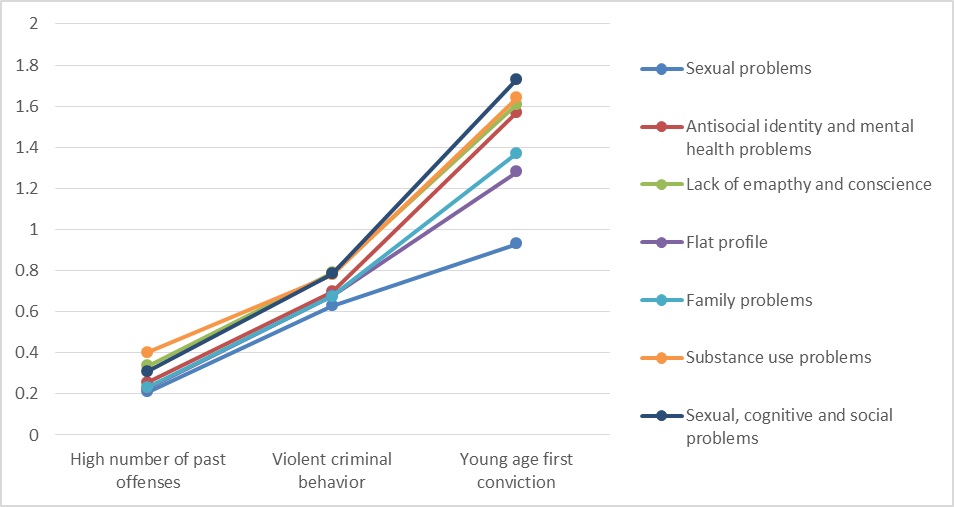

Supplement: Supplementary file 5 — Additional file 5. Item functioning of the seven subgroups on the factor Antisocial behavior. Item functioning of the seven subgroups on the factor Sexual problems. Item functioning of the seven subgroups on the factor Family background. Item functioning of the seven subgroups on the factor Mental health problems. Item functioning of the seven subgroups on the factor Substance use. Item functioning of the seven subgroups on the factor Conscience and empathy. Item functioning of the seven subgroups on the factor Cognitive and social skills. Item functioning of the seven subgroups on the factor Social network. Item functioning of the seven subgroups on the factor Offenses. [file 13034_2017_201_MOESM5_ESM.docx]
